# Editorial: Excessive internet use and its impact on mental health

**DOI:** 10.3389/fpubh.2024.1473656

**Published:** 2024-08-14

**Authors:** Aleksandar Višnjić, Kivanç Kök, Zorica Terzić-Šupić, Miodrag Stanković

**Affiliations:** ^1^Faculty of Medicine, University of Niš, Niš, Serbia; ^2^Center of Analysis, Planning, and Organization of Health Care, Institute of Public Health of Niš, Niš, Serbia; ^3^Department of Biostatistics and Medical Informatics, International School of Medicine, Istanbul Medipol University, Istanbul, Türkiye; ^4^Research Institute for Health Sciences and Technologies (SABITA), Istanbul Medipol University, Istanbul, Türkiye; ^5^Faculty of Medicine, University of Belgrade, Belgrade, Serbia; ^6^University Clinical Centre of Niš, Clinic for Mental Health, Niš, Serbia

**Keywords:** social networking, online shopping, online gambling, internet-related addictions, mental health, behavioral addictions

The various forms of Internet use, including social networking, online shopping, gambling, and gaming are products of the time in which we live and represent an integral part of how we conduct and engage in social interaction. Internet platforms not only facilitate daily connection and efficient exchange of important information, but also provide educational functions and raise awareness of problems faced by individuals and societies globally. However, excessive use of Internet-related content has also increasingly been linked with mental health issues and aspects, such as addictive behavior, which is a central theme of this Research Topic.

Within this thematic unit, 51 papers were received, of which 20 were accepted for publication. It should be emphasized that distribution of the published articles by type was as following: 17 Original research, 2 Brief Research Report and 1 Hypothesis and Theory. The reviewers, who are distinguished scholars, helped a lot to successfully accomplish this Research Topic, and we thank them on this occasion as well.

The novel approaches, rigorous research methodologies and statistically significant findings, which are presented under this Research Topic, altogether speak in favor of the fact that Excessive Internet Use is quite problematic nowadays and that more care should be taken in how to deal with it. Although any internet-related addiction is not specifically identified in the Diagnostic and Statistical Manual of Mental Disorders (DSM-5) or International Classification of Diseases (ICD-11), the growing body of literature suggest it shares a common underlying etiological framework with other (substance or behavioral) addictions.

In this regard, the connections between the excessive use of the Internet (especially social networks) with pronounced occurrences of various symptoms of mental health, as well as health in general, were unequivocally established.

On other hand, it should be noted that some research showed that Internet use significantly enhances people's happiness (Sun et al.). Relatedly, it should be noted that this article was concerning older adults. In another cohort of the older adults in somewhat extensive Chinese study by Mu et al., it was shown that time spent on the Internet significantly reduces depressive and melancholic states. From this perspective, the two mentioned studies appear to be in line with regard to the reported, somewhat positive impact on mental health.

On the contrary, in a recent survey in the Republic of Serbia, which was based on a representative sample, it was found that excessive use of the Internet was significantly associated with obesity, psychological disturbances and social maladjustment (Novaković et al.).

Furthermore, Internet addiction has emerged as a serious concern, particularly among university students, affecting academic performance and having significant psychological implications. Nevertheless, even the “athlete” university students were shown to be at increased risk for depressive symptoms and Internet addiction and decreased healthy lifestyle behaviors, which was reported by the remarkable survey by Çelik and Haney. However, it was illustrated by Cai et al. that physical exercise, gender, and academic year have a significant impact on Internet addiction among university students. In their study, freshmen students were shown to be more susceptible to experiencing Internet addiction.

Anyway, more evidences were obtained that mobile phone addiction is a risk factor for insomnia symptoms and that physical activity had (as it was expected) a significant moderating effect between mobile phone addiction and social anxiety (Wang J. et al.).

Furthermore, moreover, significant associations were observed between smartphone addiction and spending more hours daily on smartphones, poor sleep quality, as well as elevated levels of stress, anxiety, and depression (Nikolic et al.; Wu et al.). Smartphone usage has witnessed an exponential rise globally, with projections estimating a staggering 7.8 billion smartphone users by 2028.

The association between social media use and health-related variables, including fruit and vegetable intake, physical activity, obesity, tobacco smoking, mental health, and stress levels among adults in the Saudi community was demonstrated by BinDhim et al.. In particular, it should be noted that the effectiveness of relapse prevention as a treatment for Internet gaming disorder was found to be superior to the usual treatment in terms of reduction of Internet gaming disorder symptoms (André et al.).

In another article, it was also shown that people who are more likely to be Internet addicted are at significant risk of suicide-related behaviors (Kang et al.). Psychological need thwarting has been shown to be closely related to problematic smartphone use, psychological distress, and perceived support (Liao et al.).

Online shopping addiction is a behavior that causes serious problems and has become increasingly prevalent in a modern society. Within the scope of the study by Erzincanli et al., it was identified that anxiety and depression positively affect online shopping addiction, whereas financial attitude has a negative effect.

Another study implemented Fear of Missing Out Scales, Loneliness Scale, Mobile Phone Addiction Index Scale, and Depression-Anxiety-Stress Questionnaire with purpose to investigate university students (Liu et al.). The results suggest that the fear of missing out significantly positively predicts mobile phone addiction. This direct effect could be mediated by depression, while the indirect effect of fear of missing out on mobile phone addiction could be moderated by loneliness. Additionally, with the use of valid instruments and accompanying appropriate statistical analysis, Wang W. et al. showed based on the sample from six universities in China that mobile phone addiction is also associated with suicide ideation and suicide attempt among university students.

Overall, it is of great importance that the research on the impact of internet usage on mental health continues and gains momentum. Comprehensive and interdisciplinary efforts toward clarification of the hidden patterns and elusive aspects on the population level as well as on the individual level are largely needed. Let it be that Internet itself does not occur as any danger for the wellbeing of each individual. However, the utilization of the Internet has become one of the most essential tools in our modern society. That is why the future research should resolve the causal relationships between Internet addiction and the psychosocial factors related to health.

